# Cyclic stretch increases splicing noise rate in cultured human fibroblasts

**DOI:** 10.1186/1756-0500-4-470

**Published:** 2011-10-31

**Authors:** Michael Uhl, Kevin Mellert, Britta Striegl, Martin Deibler, Markus Lamla, Joachim P Spatz, Ralf Kemkemer, Dieter Kaufmann

**Affiliations:** 1 Institute of Human Genetics, University of Ulm, Albert Einstein Allee 11, D 89070 Ulm, Germany; 2 ZWE Biomaterials, Max Planck Institute for Intelligent Systems, Heisenbergstrasse 3, D 70569 Stuttgart, Germany; 3 Department New Materials and Biosystems, Max Planck Institute for Intelligent Systems, Heisenbergstrasse 3, D 70569 Stuttgart, Germany; 4 Biophysical Chemistry, University of Heidelberg, INF 253, D 69120 Heidelberg, Germany

## Abstract

**Background:**

Mechanical forces are known to alter the expression of genes, but it has so far not been reported whether they may influence the fidelity of nucleus-based processes. One experimental approach permitting to address this question is the application of cyclic stretch to cultured human fibroblasts. As a marker for the precision of nucleus-based processes, the number of errors that occur during co-transcriptional splicing can then be measured. This so-called splicing noise is found at low frequency in pre-mRNA splicing.

**Findings:**

The amount of splicing noise was measured by RT-qPCR of seven exon skips from the test genes *AATF*, *MAP3K11, NF1, PCGF2*, *POLR2A *and *RABAC1*. In cells treated by altered uniaxial cyclic stretching for 18 h, a uniform and significant increase of splicing noise was found for all detectable exon skips.

**Conclusion:**

Our data demonstrate that application of cyclic stretch to cultured fibroblasts correlates with a reduced transcriptional fidelity caused by increasing splicing noise.

## Research hypothesis

Eukaryotic cells sense the physical properties of their microenvironment by translating mechanical forces into biochemical signals. This mechanotransduction and its triggered biological responses are crucial for the regulation of many important cellular functions [1-4]. Mechanical forces are also transmitted to the nucleus through the cytoskeleton by extra- or intracellular force generation [5-7]. Even though the nucleus is suggested to be stiffer than the surrounding cytoskeleton [8,9], extracellular forces and strain result in clearly detectable deformations of the nucleus [5,10] that can in turn induce conformational changes in chromatin organization and transcriptional regulation [11-14].

A common experimental approach permitting to investigate the effects of mechanical forces on cells *in vitro *is cyclic stretching of cultured cells plated on an expandable elastomeric substrate coated with extracellular matrix components such as fibronectin. The cells dynamically align their cell bodies and cytoskeletons in a direction perpendicular to the strain [15]. The mean cell orientation changes exponentially with a frequency-dependent characteristic time from 1 to 5 h [16]. Mechanisms involved in force-induced cellular reorganisation are focal-adhesion sliding, RhoA activation and the actomyosin machinery, whereas the process seems to be largely independent of the dynamic microtubule network [17,18]. Together with the cells, their nuclei also become deformed [16,19]. Numerous *in vitro *studies have shown that cyclic stretching alters the expression of several genes depending on the type of mechanical loading, stretching magnitude, frequency and duration [20]. However, it is yet unknown whether the precision of nucleus-based processes is also influenced by cyclic stretching.

One method to determine the precision of nucleus-based processes is the detection of errors that occur during splicing (termed splicing noise) [21]. A critical step in co-transcriptional splicing is the recognition and correct pairing of the 5' and 3' splice sites of the pre-mRNA by the spliceosome. The co-transcriptional assembly of the spliceosome in a stepwise manner around the splice site junctions requires the activity of several protein factors as well as five snRNAs [21,22]. Splicing noise occurs if this process is disturbed, resulting for example in transcripts lacking one or more cassette exons. It was first detected in selected genes [22-26] and later suggested to be a more general process [27]. More recently, it was shown in a genome-wide approach to occur at low frequency in almost all genes, with splicing noise rates of approximately 0.7% for the average intron [28]. *In vitro*, splicing noise rates can be increased artificially by inhibiting the nonsense-mediated mRNA decay (NMD) or by culturing the cells at 20°C (cold shock) [22-24,26].

In this work, splicing noise rates were investigated in cultured primary human fibroblasts exposed to uniaxial cyclic tensile strain with periodic alternation of the stretch direction every two hours. In the following, this condition was termed altered uniaxial cyclic stretch leading to continuous mechanical deformations of the cells. The amplitude and frequency of the cyclic stretch is related to the periodic strain induced by the pulsatile deformation of blood vessels [16].

A reliable and established method to measure splicing noise rates is the relative quantification of erroneous splice variants, such as variants lacking one cassette exon, compared to the wildtype product by RT-qPCR [22,25]. Seven exon skip variants which have been found to be expressed in human fibroblasts were chosen out of several test genes: *AATF *(exon 3), *MAP3K11 *(exon 9), *NF1 *(exons 38 and 39), *PCGF2 *(exon 10), *POLR2A *(exon 23) and *RABAC1 *(exon 4). The detection method was validated by measuring increased splicing noise rate in cultured fibroblasts treated with cold shock.

Our study demonstrates that cyclic stretching in human fibroblasts is correlated to a reduced fidelity of a nucleus-based process by increasing the splicing noise rate in several genes.

## Methods

### Cell culture and preparation of the substrates

Tissue processing and preparation of human fibroblasts was performed as described [26]. Biopsies from two healthy Caucasian male donors, K14 and K15 aged 11 and 9 years, were obtained from the prepuce. The research was carried out in compliance with the Helsinki Declaration, obtained written ethics approval from the ethics committee (Ethikkommission Universität Ulm, A 185/09) and written informal consent from all participants and their parents. Fibroblasts of early passages were cultured in Dulbecco's modified eagle medium with 10% fetal bovine serum at 37°C and 5% CO_2 _on cell culture flasks or polydimethylsiloxane (PDMS) substrates (Sylgard 184, Dow Corning, Midland Michigan, USA). The proliferation of cells was measured as described [29]. For cold shock, fibroblasts were cultured for 24 h at 20°C [26]. In the stretching and control experiments, the cells were cultured on PDMS substrates with an elastic modulus of about 1 MPa (data not shown). The chamber-like formed substrates with an adhesion surface of 20 × 20 mm were produced as described [16]. In order to improve adhesion of the cells to the hydrophobic PDMS gel, chamber surfaces were treated with 70% Ethanol, washed with phosphate buffered saline and then coated with fetal bovine serum for 1 h at 37°C. Followed by two wash steps with phosphate buffered saline, the chambers were filled with cell culture medium prior to the seeding of the cells. Seeding was performed 48 h before the stretching experiment with a constant number of cells (n = 40 000 per chamber). The adhesion and distribution of the cells was examined 12 h later via light microscopy. Chambers with equally distributed cells were selected for cyclic stretching experiments to minimize effects of various cell densities.

### Altered uniaxial cyclic stretching of cells

Uniaxial strain was applied with 8% amplitude and a frequency of 1 Hz with a change in stretch direction from x- to y-direction every two hours. The mechanical stimulation was performed by a customized stretching device equipped with two brushless servomotors (Faulhaber, Schoenaich, Germany) in an incubator at 37°C and 5% CO_2_. Stretching of the cells was performed overnight (18 hours) with the motors connected to a personal computer and controlled by Faulhaber Motion Manager 4 and Image Pro Plus 6 software (Media Cybernetics, Bethesda, USA) respectively. These conditions have previously been shown to be tolerable and non-toxic for human fibroblasts [16]. Cells cultured on an unstretched PDMS substrate were used as control in the same incubator until the end of the experiment. Immediate RNA isolation out of the stretched cells and unstretched control cells followed.

### RNA isolation and cDNA synthesis

Isolation of total RNA was accomplished using the RNeasy Mini Kit (Qiagen, Hilden, Germany) according to suggestions of minimum standards guidelines for fluorescence based qPCR experiments [30]. Both the stretched substrate and the control substrate were put on ice immediately after the stretching experiment was stopped, and the cells were lysed through incubation with the kit's lysis buffer containing 1% 2-mercaptoethanol for 2 minutes. After using a cell scraper to scratch the lysed cells off the PDMS surface, the lysate was transferred to a Qiashredder spin column according to the manufacturer's protocol. Amount of RNA was determined by Nanodrop. The quality of RNA isolation was assessed by gel-electrophoretic separation of RNA samples and PCR on RNA to detect DNA contaminations using intronic *NF1 *primers. Reverse transcription of RNA to produce cDNA was performed using random hexamers together with the Superscript III Kit (Invitrogen, Karlsruhe, Germany).

### Tested erroneous splice products

The following criteria were applied to select test genes with potential erroneous splice products in the form of one-cassette exon skips detectable by RT-qPCR [22]: First, the genes should facilitate detection of transcripts due to their ubiquitous and relatively high expression. Second, the gene of interest should only have one known splice form listed in Ensembl database http://www.ensembl.org. Taking this as an indicator for a constitutively spliced transcript, only the basic failure rate of a certain splicing event should be detected, rather than the abundance of an alternatively spliced product. Third, resulting transcript sequences of potential exon skips had to be checked for possible genomic and mRNA pendants. In-frame exon skips were preferentially selected to overcome possible effects of NMD, as well as exon skips with longer upstream introns, which presumably raise the rate of exon skipping [21,27]. According to these criteria, 18 exon skips in 12 genes were selected and the detection of the skip products was established as described below. After verifying the specificity of the primers with various templates (see below), five exon skips from five genes were chosen exclusively for this study (Table 1): *AATF *(Apoptosis antagonizing transcription factor, exon 3), *MAP3K11 *(Mitogen-activated protein kinase kinase kinase 11, exon 9), *PCGF2 *(Polycomb group ring finger 2, exon 10), *POLR2A *(Polymerase (RNA) II (DNA directed) polypeptide A, exon 23) and *RABAC1 *(Rab acceptor 1, exon 4). Moreover, two out of frame exon skips from *NF1 *(Neurofibromin, exons 38 and 39), although not fitting the search criteria, were selected, since they already had been established in previous studies [25].

**Table 1:** Structural data of the investigated transcripts.

Gene	Exon length	Intron length	
		**Upstream**	**Downstream**

*AATF *exon 3	411	2480	473

*MAP3K11 *exon 9	375	5992	765

*NF1 *exon 38	341	1246	2456

*NF1 *exon 39	203	2456	4339

*PCGF2 *exon 10	81	2182	489

*POLR2A *exon 23	105	1582	86

*RABAC1 *exon 4	102	1166	83

Genes and skipped exons with exon and intron lengths (in nt).

### Measuring splicing noise rates in qPCR

To specifically amplify the selected exon skip products in the cDNA samples, primers whose 3' ends overlap the newly generated exon-exon boundaries by 2 to 7 nucleotides were used (Table 2) [31]. The specificity of the primers was verified in PCR (greater 45 cycles) by using genomic DNA as a template as well as 60 mer wildtype oligonucleotides (1 amol) that reproduce the regular exon-exon boundaries from the wildtype transcripts in order to detect possible mispriming of the skip primers and undesired amplification of the wildtype transcripts. Amplified products in qPCR should only be detectable with cDNA (100 ng RNA equivalent/PCR) as template, while showing no products on genomic DNA and 60 mer wildtype oligonucleotides, respectively. The efficiencies of the primers were determined via standard curves using oligonucleotides representing the sequences of the wildtype or the exon skip products. For relative quantification, both wildtype and exon skip products were measured using the 7900 HT Fast Realtime PCR-System (Applied Biosystems, Foster City, USA) together with the QuantiTect SybrGreen kit (Qiagen, Hilden, Germany) for detection. The splicing noise frequencies were calculated as relative amounts of the exon skipped product to the wildtype products being represented by cycle threshold counts (CT-value) in RT-qPCR (ΔCT). The measured cycle threshold counts were efficiency corrected to simulate a primer efficiency of 100% (splicing noise = (1/2^ΔCT^) * 100%; ΔCT = log_2_(_Skip_E^CT(Skip)^) - log_2_(_WT_E^CT(WT)^) which represents the efficiency corrected CT-value of the misspliced product - the corrected CT-value of the wildtype product; E = primer efficiency) [31]. The detection method was validated by the measurement of exon skips in cold shock treated cultured fibroblasts. Results are presented as mean ± SD. Statistical analysis was performed with a two sample paired t-Test while P < 0.05 was defined as statistically significant.

**Table 2:** Primers used to detect skip and wildtype transcripts.

Name	Exon	Sequence 5'-3'	P	AT
AATF S3 H	2-4	CCAGGATCGTCTGCACTG	96	59.5

AATF S3 R	4	AACATCTGGTTGAGGAAGCTG	96	59.5

AATF WT H	2	GACACGGACAAAAGGTATTGC	122	59.5

AATF WT R	3	TCTCCAGACCCTTCCTCATC	122	59.5

MAP3K11 S9 H1	8-10	CCAGCACTCAATGGAGGC	149	59.5

MAP3K11 S9 R	10	AAGCTCCAGGGATCAATGC	149	59.5

MAP3K11 WT H	7	GGAGGACTCAAGCAATGGAG	120	59.5

MAP3K11 WT R	8	AGGTACCATGTGGCTTCGTC	120	59.5

NF1 S38 H	37-39	AGACACCAAAGTTTCTATTAAAGTCAGCT	62	60

NF1 S38 R	39	GGTACAAGTTAAGGCACACAGAAGATTA	62	60

NF1 S39 H	38-40	TCTGACCCGAGTTTACGGTATTG	72	60

NF1 S39 R	40	AGATTTGACAGCCATGGAGTCAT	72	60

NF1 WT H	38	GCAGTTCTGACCCGAGTTTACG	99	60

NF1 WT R	39	ATGTCTCTAGTAACTGGCCCTCGAT	99	60

PCGF2 S10 H1	9-11	CCCAGCAAGTACAAGAACGG	139	60

PCGF2 S10 R1	11	CGCTGACTGACTCACACTCG	139	60

PCGF2 WT H	9	AAGTTTCTCCGCAACAAGATG	100	60

PCGF2 WT R	10	CGATGTCCATGAGGGTGTAG	100	60

POLR2A S23 H	22-24	GAGAACAAGATGCAAGAGGTGTAC	88	59.5

POLR2A S23 R	24	CCTTGAATTCCCCATCCTC	88	59.5

POLR2A WT H	22	GTTTTGGTGACGACTTGAACTG	145	59.5

POLR2A WT R	23	CGCAGGAAGACATCATCATC	145	59.5

RABAC1 S4 H	3	CGTCCCCTATGTTGCTGGTG	107	63

RABAC1 S4 R2	3-5	CACCAGGGTGGCTCCAAAG	107	63

RABAC1 WT H1	2	GCAACTATGTGTTCGTGTTCCTG	148	61

RABAC1 WT R1	3-4	TCTCGGCCAAAGAGCACA	148	61

Primers for detection of erroneously spliced (S) and wildtype (WT) *AATF*, *MAP3K11*, *NF1*, *PCGF2*, *POLR2A *and *RABAC1 *transcripts respectively, their position related to the exons (Exon), sequence, length of the amplified product (P in bp) and annealing temperature (AT in °C). The applied concentrations in qPCR were 200 nM.

## Results

### Reliable measuring of splicing noise rates in RT-qPCR

**Figure 1:**
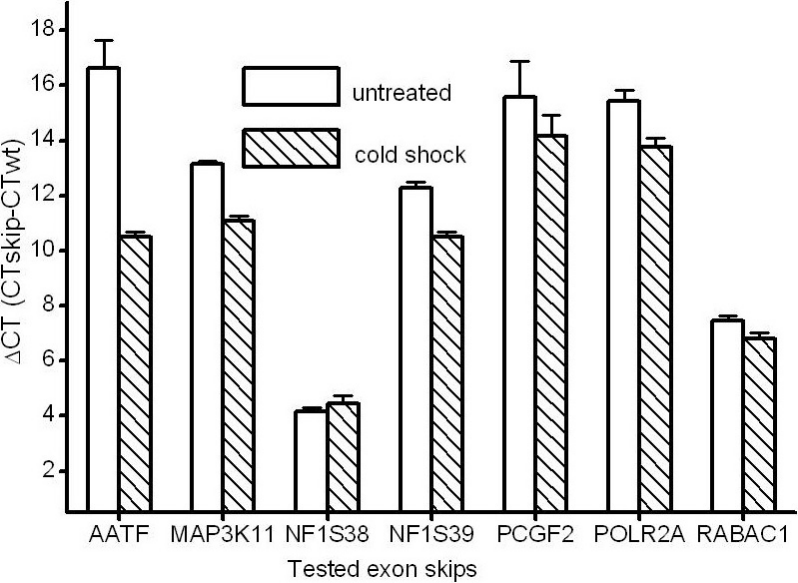
**Cold shock increases splicing noise rates in cultured fibroblasts**. ΔCT of CT of the exon skip transcripts AATF-Δ3 (AATF), MAP3K11-Δ9 (MAP3K11), NF1-Δ39 (NF1S38), NF1-Δ38 (NF1S39), PCGF2-Δ10 (PCGF2), POLR2A-Δ23 (POLR2A), RABAC1-Δ4 (RABAC1) and of the CT of the wildtype transcripts in cDNA from untreated fibroblasts and fibroblasts treated with cold shock (differences in SD). Reduced ΔCT indicates an increased splicing noise rate.

The detection of the five in-frame exon skips AATF-Δ3, MAP3K11-Δ9, PCGF2-Δ10, POLR2A-Δ23 and RABAC1-Δ4 was established in RT-qPCR. All exon skip primers were solely capable of amplifying their corresponding exon skip products in cDNA samples of cultured primary human fibroblasts in standard PCR as shown by sequencing of the PCR products. Moreover, all exon skip primers passed the specificity tests with no products on genomic DNA or wildtype 60 mer oligonucleotides, while fibroblast cDNA as a template led to the predicted products. Additionally two previously established out of frame exon skips, NF1-Δ39 and NF1-Δ38, were investigated [25]. RT-qPCR measurement of the 7 exon skips in a cDNA sample from cultured fibroblasts (K14) yielded differences regarding their relative abundance represented as ΔCT values (Table 3). An estimate of the percentage of the amount of the exon skip product relative to the wildtype product results in differences between 0.001 to 5.609%. To test whether a doubling of the exon skip rates could also be measured in RT-qPCR, fibroblasts (K15) plated on culture dishes were exposed to cold shock. This resulted in a significant increase in the relative splicing noise rates (ΔCT) of the exon skips in these cells (P = 0.024) (Figure 1). These results demonstrate that the RT-qPCR method is capable of detecting differences in the splicing noise rates of the established exon skips.

**Table 3:** Relative occurrence of the exon skip transcripts (ΔCT) in cultured fibroblasts.

Gene/exon	CT (WT)	CT (skip)	ΔCT	(%)
AATF-Δ3	23.022 ± 0.130	39.630 ± 1.041	16.607 ± 1.049	(0.001%)

MAP3K11-Δ9	24.228 ± 0.095	37.384 ± 0.041	13.156 ± 0.103	(0.010%)

NF1-Δ38	23.221 ± 0.083	27.377 ± 0.096	4.156 ± 0.127	(5.609%)

NF1-Δ39	22.615 ± 0.045	34.883 ± 0.220	12.267 ± 0.225	(0.020%)

PCGF2-Δ10	23.214 ± 0.201	38.793 ± 1.311	15.579 ± 1.326	(0.002%)

POLR2A-Δ23	24.408 ± 0.205	39.826 ± 0.350	15.419 ± 0.405	(0.002%)

RABAC1-Δ4	22.304 ± 0.131	29.750 ± 0.123	7.446 ± 0.180	(0.573%)

CT of the skip trancripts of AATF-Δ3, MAP3K11-Δ9, NF1-Δ38, NF1-Δ39, PCGF2-Δ10, POLR2A-Δ23 and RABAC1-Δ4 in relation to CT of the wildtype transcripts (number of measurements n = 3, for exon skip n = 4) in cultured fibroblasts (K14). CT values for wildtype (WT), the exon skip transcripts (skip) and the difference (ΔCT) (SD) and the percentage (%) of the amount of the exon skip product relative to the wildtype product.

### Cyclic stretching of primary fibroblasts

The viability of the primary fibroblasts plated on the PDMS surface was observed by comparing the proliferation rates on both the PDMS surface and the culture flask surface showing no alteration. The cellular response to cyclic stretching was controlled by applying uniaxial cyclic strain (5 h, stretching frequency 1 s^-1^, amplitude 8%) whilst observing the cells by phase contrast light microscopy. A perpendicular orientation of cells relative to the stretching direction was observed. In unilaterally stretched cells the nuclei were deformed by about 4.8% as determined by measuring the circularities and the aspect ratios of the marked nuclei (data not shown). To test whether altered unilateral cyclic stretching with the applied parameters was tolerated by the primary fibroblasts, the RNA content of stretched cells and unstretched controls was compared. As a result, the total yield of RNA was within 1.6 and 4.1 μg/10^5 ^cells for both conditions, indicating that the applied stretching is a tolerable condition.

### Increased splicing noise rates in altered uniaxial cyclically stretched cells

**Figure 2:**
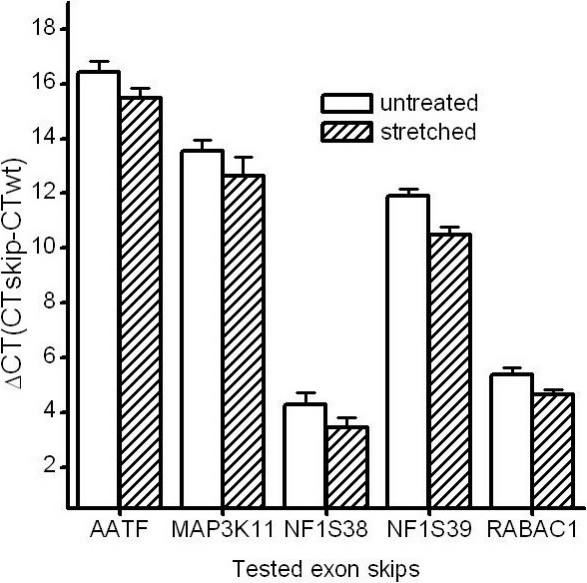
**Altered uniaxial cyclic stretching of fibroblasts doubles splicing noise rates**. ΔCT of CT of the exon skip transcripts AATF-Δ3 (AATF), MAP3K11-Δ9 (MAP3K11), NF1-Δ39 (NF1S38), NF1-Δ38 (NF1S39) and RABAC1-Δ4 (RABAC1) to the CT of the wildtype transcripts in cDNA from fibroblasts cultured on PDMS (untreated) and treated by cyclic stretching (stretched) are shown.

Altered uniaxial cyclic strain was applied for 18 hours and immediately followed by RNA isolation, cDNA synthesis and qPCR measurements. In cDNA samples of the unstretched control cells, the detection and evaluation of splicing noise rates was successful in five out of seven exon skips, while PCGF2-Δ10 and POLR2A-Δ23 were not detectable. The splicing noise rates between the other five investigated exons differed similarly to the ones in cells cultured on normal culture dishes. No significant differences in splicing noise rates of the exons were detected between unstretched cells cultured on PDMS substrates or normal culture dishes (P = 0.22). In contrast, when comparing stretched cells to unstretched controls, the splicing noise rates in stretched fibroblasts approximately doubled in all of the five tested exons (Figure 2) and this rate change was shown to be statistically significant (P = 0.0005). Furthermore, PCGF2-Δ10 was only detectable in the stretched cells. Given the fact that the measured differences in splicing noise rates result from the transcription of four different genes and additionally that PCGF2-Δ10 was only detectable in stretched cells, it can be concluded that splicing noise rates increase systematically upon cyclic stretching in cultured fibroblasts.

## Discussion

In this study, a uniform and significant increase in splicing noise was found for all detectable exon skips in fibroblasts treated by cyclic stretching. Similarly doubled splicing noise rates were found previously in spinal muscle atrophy (SMA) patient fibroblasts compared to control fibroblasts [22]. SMA is commonly caused by the deletion of one of the two copies of the *SMN *gene, resulting in an insufficient expression of SMN, which is involved in the assembly of spliceosomal components, explaining the observed uniform increase in splicing noise rates. Therefore, it has to be assumed that consistently doubled splicing noise rates indeed exert an *in vivo *effect with presumable clinical relevance. Given a certain basic error rate per splicing reaction, it can further be assumed that genes with a higher number of introns bear less functional wild type transcripts and therefore should be more affected by uniformly increased splicing noise rates. This could be especially meaningful if a tightly balanced gene dose is inevitable, such as for tumor suppressor genes [26]. On the other hand, several proteins involved in the RNA surveillance mechanism NMD which has to cope with splicing noise also play roles in cell cycle progression or telomerase maintenance, and NMD is becoming more and more understood as a regulated process which additionally alters the expression of alternatively spliced isoforms as well as being involved in tumorigenesis [32]. It could thus be interesting to investigate the influences of increased splicing noise rates on NMD in future studies.

Most human cells *in vivo *are continuously exposed to external forces as per those due to the periodic strain induced by the pulsatile deformation of blood vessels. Cyclic stretching of cultured fibroblasts on PDMS substrates can be one experimental approach to investigate cellular responses to such periodic forces and has been applied in various studies investigating the mechanoregulation of gene expression [20]. Nuclear deformations can influence transcriptional regulation [12,33]. However, the mechanism by which nucleus deformation induces altered gene expression is still unknown. It has been suggested that nucleus deformation influences the positioning of chromosomes mechanically linked to components of the inner nuclear membrane [34]. Mechanical forces thus may also influence chromatin structure and in particular nucleosome positioning [35]. Varying stretching amplitudes and frequencies have been observed in a previous study concerning the cell's dynamic reorientation behaviour [16]. In this regard, it would be interesting in a follow-up study to check whether variations of the applied stretching parameters correlate with the resulting amount of splicing noise. Moreover, the recent identification of the two transcriptional regulators YAP and TAZ should give additional molecular insight because of their involvement in mediating biological responses to mechanical inputs such as variations of extracellular stiffness or changes in cell shape [36].

Here we chose to investigate a different effect of cyclic stretching on nuclear function, namely the precision of a nucleus-based process. We analysed the error rates in co-transcriptional splicing of several test genes as detected by RT-qPCR. This approach was proved to be a sensitive and reliable method for detecting differences in splicing noise rates in small amounts of primary cells [22,31]. As demonstrated recently in a genome wide approach, splicing noise is a general process [27]. Therefore, test genes and exons could be selected more or less stochastically.

The quite uniform increase in splicing noise we found in these few genes most probably represents a general trend. However, a more systematic and expensive approach will be helpful to further confirm the effects of cyclic stretching on splicing noise rates such as a genome wide deep cDNA sequencing.

As to the cause of the increased splicing noise rates in cyclically stretched cells, a possible direct effect influenced by the mechanical deformation of the nucleus still has to be further validated in the following. One possible strategy to support this idea would be to disrupt cytoskeletal structures physically linking the nucleus to the periphery. In addition, it would be informative to find out whether the observed effects on transcription fidelity are related to the activation of some heat shock proteins such as hsp-72 or hsp-90 [37,38] or to the expression of YAP or TAZ [36]. Until now, there are no evidences that the expression of these genes influences splicing noise rates. Finally, the observed general increase in splicing noise rates in cyclically stretched cultured fibroblasts raises the question of its physiological relevance. Minding the fact that some body cells are subjected to the periodic strain, the described experimental conditions could indeed resemble the cell's physiological environment better than standard cell culture conditions. Hence, further observation of splicing noise rates in other cell types such as endothelial cells, could give greater insight into co-transcriptional fidelity in different cell types or even tissues.

## Conclusions

In summary, the described work represents the first examination on the effects of cyclic stretching of cultured cells on splicing noise rates. In the selected genes, a uniform significant increase was found in cultured human fibroblasts.

## Competing interests

The authors declare that they have no competing interests.

## Authors' contributions

Authorship credit is based on substantial contribution to conception and design, or acquisition of data, or analysis and interpretation of data (MU, KM, BS, RK, DK), drafting the article or revising it critically for important intellectual content (MU, KM, MD, ML, JPS, RK, DK). All authors read and approved the final manuscript. All authors declare there are no financial, personal, or professional interests that could be construed to have influenced the paper.
